# Supplementary material for: Fibroblast state switching orchestrates dermal maturation and wound healing
Source: Mol Syst Biol. 2018 Aug 29;14(8):e8174. doi: 10.15252/msb.20178174 (PMC6113774; doi:10.15252/msb.20178174)
Supplement: Supplementary file 1 — Expanded View Figures PDF [file MSB-14-e8174-s001.pdf]

Expanded View Figures

Figure EV1. Characterising dermis expansion and gene expression changes during mouse development (related to Fig 1).

A Modelling dermal fibroblast cell divisions during development (adapted from Rognoni *et al*, 2016). Predicted number of dermal fibroblast divisions during the transition from embryonic (E17.5) to neonatal (P2) and neonatal (P2) to adult (P50) mouse. Height, length and dermis diameter were measured (n = 3 mice per time point and gender), and the dermis volume was estimated by representing the mouse trunk as a cylinder. Cell densities were obtained from Fig 1A, and cell number at E17.5 (N_E), P2 (N_N) and P50 (N_A) was estimated by multiplying cell density and dermis volume. The predicted cell division rate is calculated by the $\log_2$ of the $N_{older}/N_{younger}$ ratio. Calculated values are shown in Table EV1.

B Representative cell cycle flow cytometry profiles for indicated time points. Note the sharp decrease in S-phase with age and the arrest in G1.

C Comparative analysis of the transcriptomics of neonatal and adult mouse fibroblasts (GSE32966). The volcano plot (left panel) illustrates the differences in fibroblast gene expression at different ages. Colour code indicates entities not statistically significantly changed (grey), statistically significant but not enriched (green) or significantly changed with fold change > 2 and P-value smaller than 0.05. Red corresponds to enriched in neonatal and blue to enriched in adult. Gene ontology (GO) analysis (right panel) of neonatal and adult fibroblasts. GO terms are highlighted in neonates (red) and adults (blue).

© 2018 The Authors

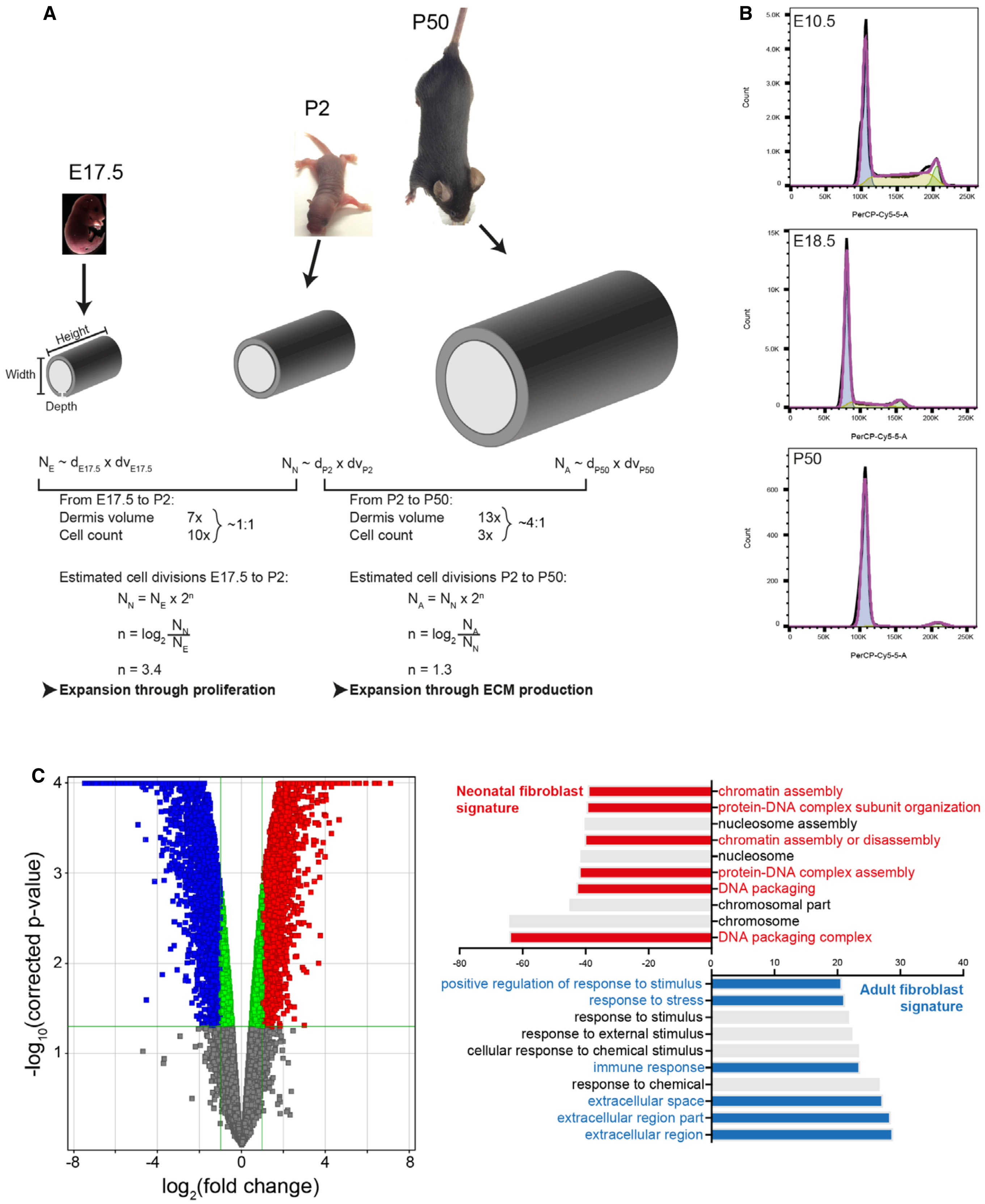

Figure EV1.

 © 2018 The Authors

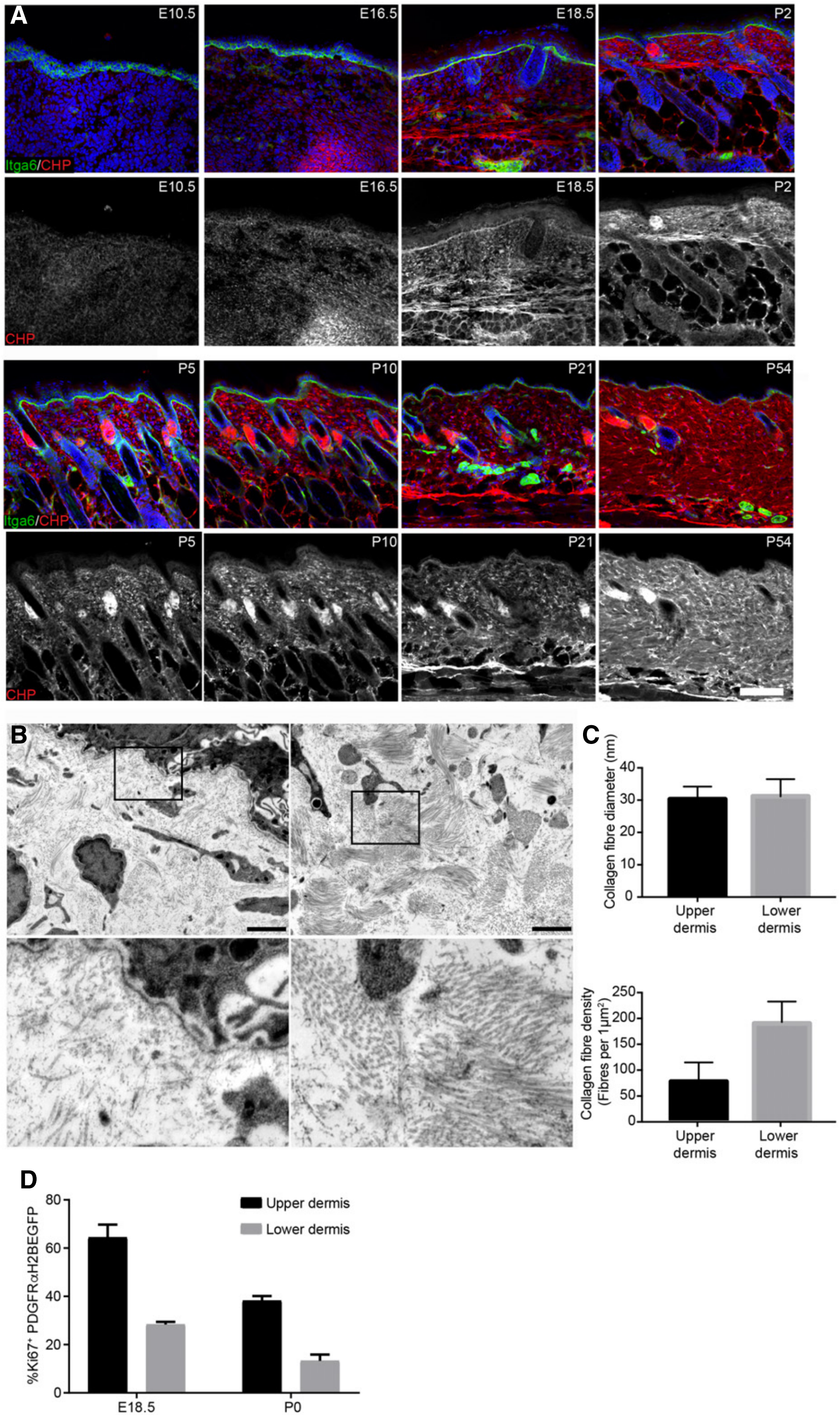

Figure EV2. Distinct collagen structures in the upper and lower neonatal dermis (related to Fig 1).

A Immunofluorescence staining of back skin sections at indicated developmental time points for Itga6 (green) and collagen (red, white) using the CHP-biotin probe. Nuclei were labelled with DAPI (blue). Note the appearance of collagen fibres after E18.5.

B TEM image of upper (papillary layer, left) and lower (reticular layer, right) dermis at P2. Boxed area is magnified below.

C Quantification of collagen fibre diameter (upper panel; n = 42 fibres) and density (lower panel) in upper and lower dermis at P2 (n = 10 areas).

D Quantification of proliferating fibroblasts (Ki67^{+} cells) in the upper and lower dermis at indicated time points (n = 3 E18.5; n = 2 P0 biological replicates).

Data information: Scale bars: 100 µm (A), 2 µm (B). Error bars represent standard deviation of the biological replicates.

© 2018 The Authors

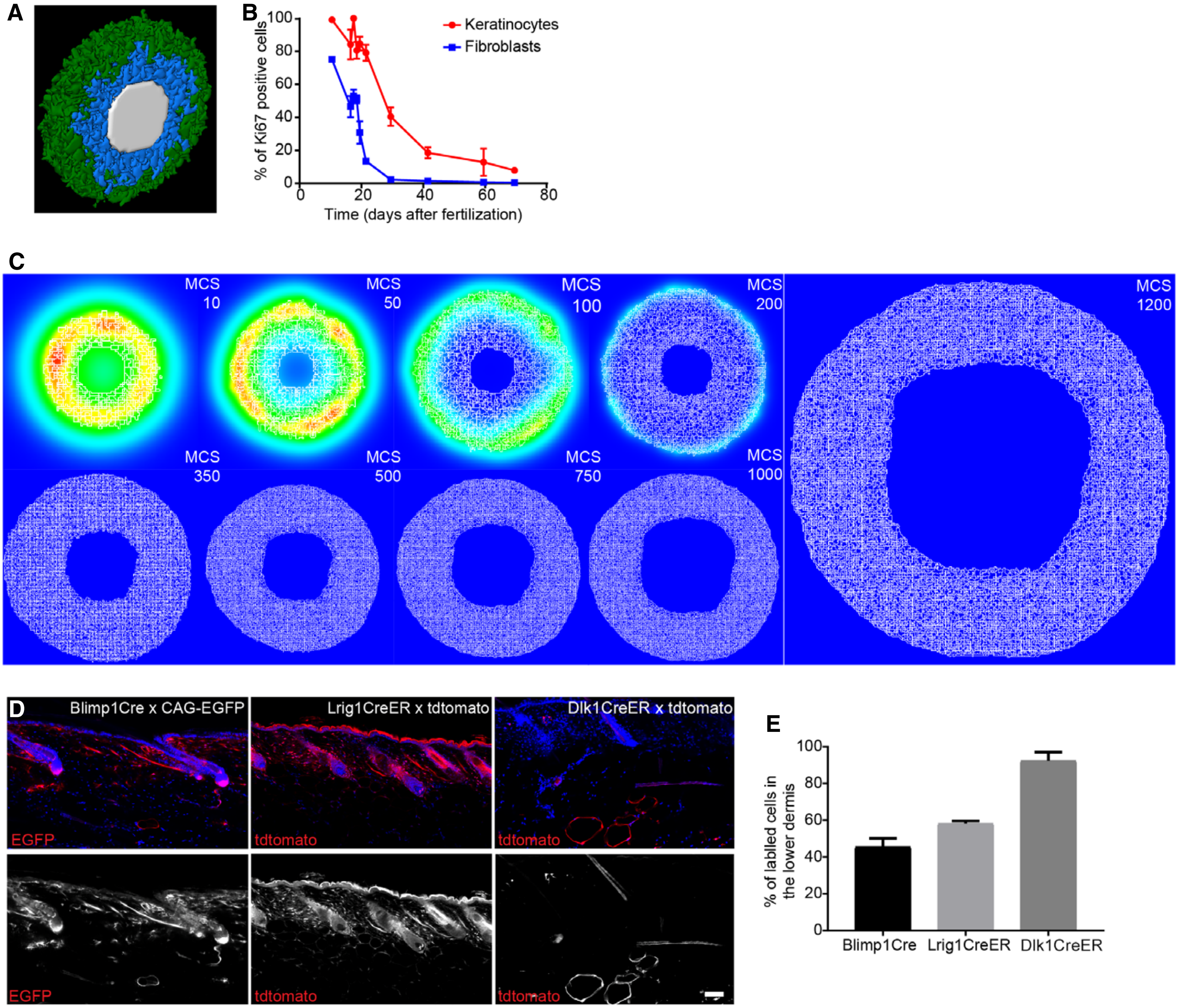

Figure EV3. Skin proliferation kinetics and *in vivo* fibroblast lineage tracing during dermal maturation (related to Fig 4).

A 3D visualisation of the simulated mouse body segment. Colour code indicates epidermis in green, proliferating fibroblasts in blue and lumen in white.

B Quantification of proliferating (Ki67-positive) keratinocytes and fibroblasts in skin over time (n = 4 for 16.5, 29.5; n = 3 for 10.5, 18.5, 21.5, 30.5, 63.5, 69.5; n = 2 for 17.5, 19.5; biological replicates). Note that the decrease in proliferation of keratinocytes and fibroblasts follows similar kinetics over time.

C Representative simulation images of the epidermal gradients at indicated MCS. Note that the signal concentrates in the immediate surroundings of the epidermal cells and decays over time.

D, E *In vivo* lineage tracing of upper (Blimp1$^+$ and Lrig1$^+$ cells) and lower dermis (Dlk1$^+$ cells) fibroblasts. (D) Immunofluorescence image of tdtomato or CAG-EGFP-labelled fibroblasts (red) with the indicated Cre lines. Nuclei were labelled with DAPI (blue). (E) Quantification of the percentage of labelled fibroblasts in the lower dermis of adult mice (> P50) (n = 2 biological replicates). Note the increased abundance of Blimp1Cre- and Lrig1CreER-labelled dermal fibroblasts in the lower dermal layer with age.

Data information: Data shown are means ± s.d. Scale bar, 100 µm.

 © 2018 The Authors

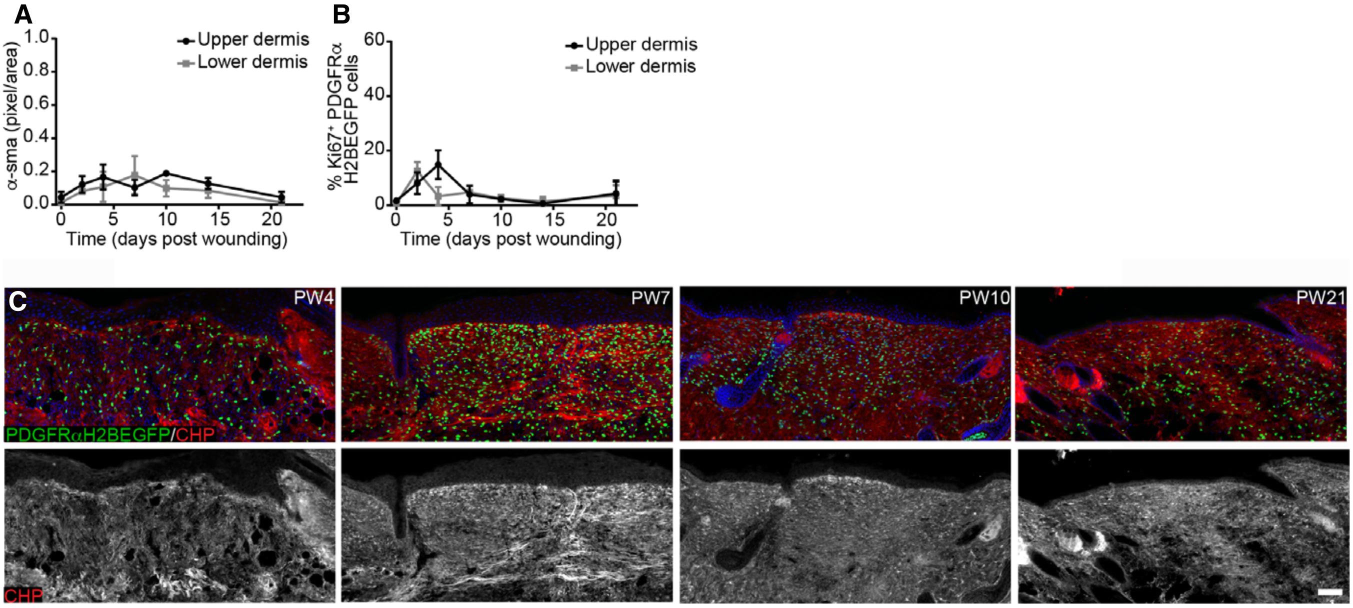

Figure EV4. Changes in fibroblast activation and proliferation outside the wound and CHP staining in the wound bed (related to Fig 5).

A Quantification of α-sma fluorescence intensity in pixels per unit area in the upper and lower dermis outside the wound bed (n = 4 PW0, PW2, PW7, PW21; n = 3 PW10, PW14; n = 2 PW4 biological replicates).

B Quantification of fibroblast proliferation in the upper and lower dermis outside the wound bed over time (n = 4 PW0, PW2, PW21; n = 3 PW4, PW10; n = 2 PW7, PW14 biological replicates).

C Immunofluorescence staining of adult PDGFRαH2BEGFP (green) wounds at indicated time points for collagen (red, white) using the CHP-biotin probe. Note that collagen fibres are appearing at PW7 in the upper and lower wound bed. Nuclei were labelled with DAPI (blue).

Data information: Data shown are means ± s.d. Scale bar, 100 μm.

© 2018 The Authors

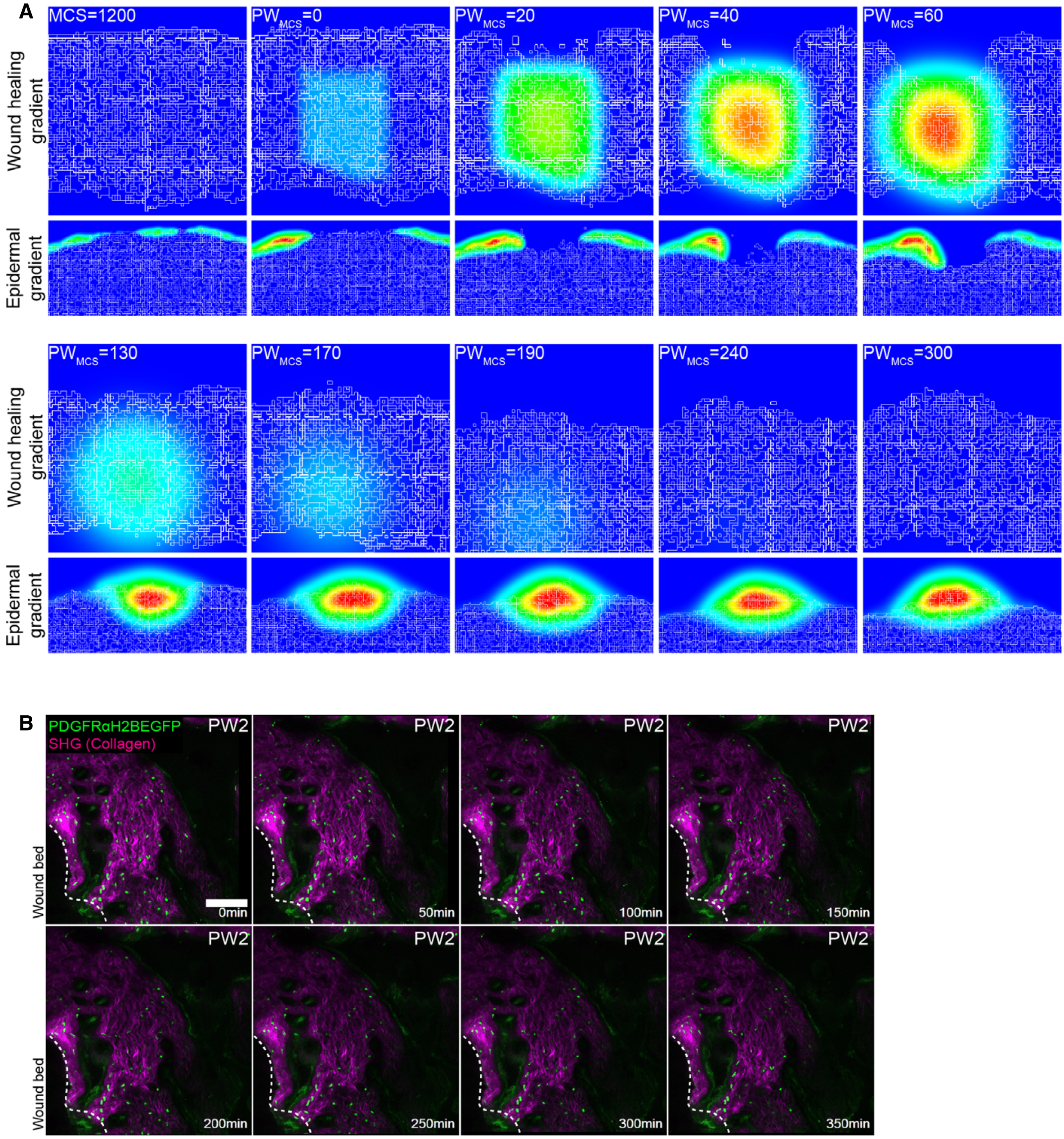

Figure EV5. Computational wound healing gradients and *in vivo* imaging of the wound bed at PW2 (related to Fig 6).

A Gradients used in the computational model. Top panel represents the wound healing gradient during the course of the simulation. Bottom panel represents the epidermal gradient in the vicinity of the wound during the course of the simulation.

B Representative time-lapse images of adult PDGFRαH2BEGFP dermal fibroblasts (green) and collagenous extracellular matrix shown with second harmonic generation (SHG) in purple 2 days after wounding. Dotted line indicates wound edge. Note that fibroblasts are not actively migrating at 2 days postwounding. Please refer to associated Movie EV5. Scale bar, 100 µm.

 © 2018 The Authors

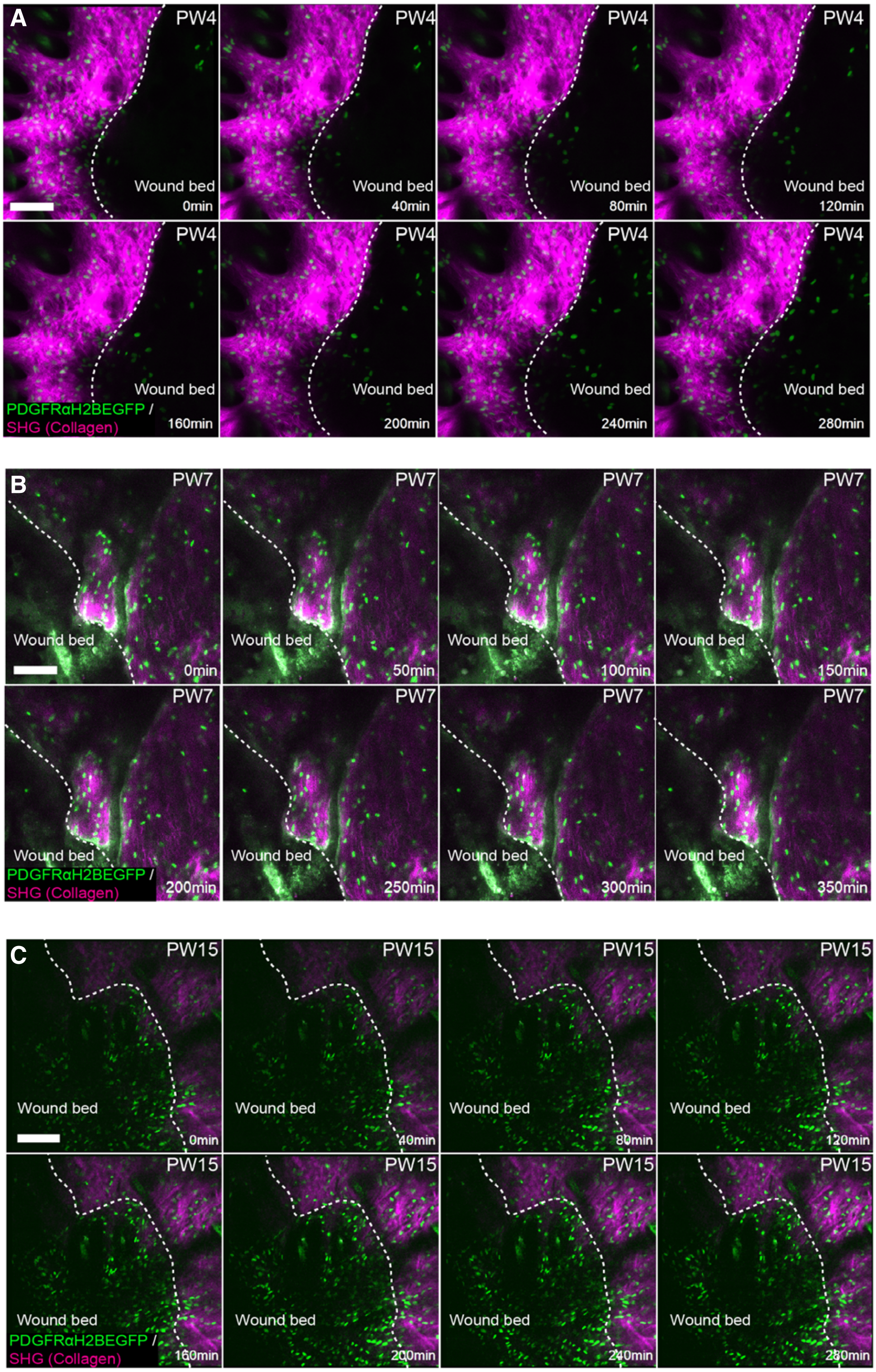

Figure EV6. *In vivo* imaging of the wound bed at later time points (related to Fig 7).

A–C Representative time-lapse images of adult PDGFRαH2BEGFP dermal fibroblasts (green) with collagenous extracellular matrix shown with second harmonic generation (SHG) in purple 4 days (A), 7 days (B) and 15 days (C) after wounding. Dotted line indicates wound edge. Note that fibroblasts are actively migrating at 4 but not at 7 or 15 days postwounding. Please refer to associated Movies EV6 and EV7. Scale bars, 100 μm.

© 2018 The Authors
